# Novel Vpx virus-like particles to improve cytarabine treatment response against acute myeloid leukemia

**DOI:** 10.1007/s10238-024-01425-w

**Published:** 2024-07-13

**Authors:** Ramya Nair, Alejandro Salinas-Illarena, Monika Sponheimer, Inès Wullkopf, Yannick Schreiber, João Vasco Côrte-Real, Augusto del Pozo Ben, Helena Marterer, Dominique Thomas, Gerd Geisslinger, Jindrich Cinatl, Marion Subklewe, Hanna-Mari Baldauf

**Affiliations:** 1 Max Von Pettenkofer Institute and Gene Center, Virology, National Reference Center for Retroviruses, Faculty of Medicine, LMU München, Feodor-Lynen-Str. 23, 81377 Munich, Germany; 2 Department of Medicine III, University Hospital, LMU, Munich, Germany; 3 Laboratory for Translational Cancer Immunology, LMU Gene Center, Munich, Germany; 4 Fraunhofer Cluster of Excellence for Immune Mediated Diseases CIMD, Fraunhofer Institute for Translational Medicine and Pharmacology ITMP, 60596 Frankfurt Am Main, Germany; 5 CIBIO-InBIO, Research Center in Biodiversity and Genetic Resources, University of Porto, 4485-661 Vairão, Portugal; 6 Department of Biology, Faculty of Sciences, University of Porto, 4169-007 Porto, Portugal; 7 BIOPOLIS Program in Genomics, Biodiversity and Land Planning, CIBIO, Campus de Vairão, 4485-661 Vairão, Portugal; 8 Institute for Clinical Pharmacology, Goethe University Frankfurt, 60590 Frankfurt Am Main, Germany; 9 Institute for Medical Virology, University Hospital, Goethe University, Frankfurt Am Main, Germany; 10 Dr. Petra Joh-Forschungshaus, Frankfurt Am Main, Germany; 11 German Cancer Consortium (DKTK) and German Cancer Research Center (DKFZ), Heidelberg, Germany

**Keywords:** SAMHD1, Vpx, VLP, Cytarabine, AML, Resistance

## Abstract

Knowledge of the molecular pathogenesis of acute myeloid leukemia has advanced in recent years. Despite novel treatment options, acute myeloid leukemia remains a survival challenge for elderly patients. We have recently shown that the triphosphohydrolase SAMHD1 is one of the factors determining resistance to Ara-C treatment. Here, we designed and tested novel and simpler virus-like particles incorporating the lentiviral protein Vpx to efficiently and transiently degrade SAMHD1 and increase the efficacy of Ara-C treatment. The addition of minute amounts of lentiviral Rev protein during production enhanced the generation of virus-like particles. In addition, we found that our 2nd generation of virus-like particles efficiently targeted and degraded SAMHD1 in AML cell lines with high levels of SAMHD1, thereby increasing Ara-CTP levels and response to Ara-C treatment. Primary AML blasts were generally less responsive to VLP treatment. In summary, we have been able to generate novel and simpler virus-like particles that can efficiently deliver Vpx to target cells.

**Supplementary Information:**

The online version contains supplementary material available at 10.1007/s10238-024-01425-w.

## Introduction

Clonal expansion of undifferentiated myeloid progenitors is characteristic for the development of acute myeloid leukemia (AML), one of the most common forms of leukemia in adults [1]. AML is highly heterogeneous due to genetic, epigenetic, and phenotypic leukemia factors [2]. Current treatment regimens typically include the nucleoside analog cytarabine (Ara-C) and the anthracycline daunorubicin, but personalized treatment options are now increasingly available and new preclinical targets have been identified [3, 4]. However, poor response and survival rates, especially in elderly patients, are still a current problem [5].

We and others recently identified the sterile alpha motif and HD domain-containing protein 1 (SAMHD1) as a biomarker for Ara-C resistance in AML patients [6, 7]. SAMHD1 hydrolyzes the active form of Ara-C, Ara-CTP, drastically reducing the cytotoxic potency of Ara-C [6]. High levels of SAMHD1 also negatively affect the clinical response to decitabine and nelarabine in AML, acute lymphoblastic leukemia, and Hodgkin lymphoma [8–10].

SAMHD1 is a deoxynucleoside triphosphate triphosphohydrolase [11] cleaving dNTPs into deoxyribonucleotides and inorganic triphosphate. It restricts replication of human immunodeficiency virus (HIV-1) [12, 13]. HIV-2 and certain simian immunodeficiency viruses (SIVs) express the viral protein X (Vpx), which depletes intracellular SAMHD1 levels via proteasomal degradation [12, 13]. Vpx is homologous to viral protein R (Vpr), which is present in all primate lentiviruses. Vpr is generally not counteracting SAMHD1-mediated viral restriction, but De Brazza's monkeys (Cercopithecus neglectus, SIVdeb strain) and mustached monkeys (Cercopithecus cephus, SIVmus strain) Vpr proteins degrade SAMHD1 [14].

Previously, we used Vpx-carrying virus-like particles (VLPs) derived from SIV, which transiently degraded SAMHD1 and increased the response rate of Ara-CTP [6]. VLPs are non-infectious, self-assembling viral proteins of various shapes, sizes, and origins [15]. Due to their inherent ability to encapsulate specific mRNAs and proteins, they are used as delivery vehicles in gene therapy and vaccine approaches [16, 17]. Because SAMHD1 plays such a critical role in dNTP homeostasis, we conducted a proof-of-concept study to genetically minimize the SIV-based VLP [6] to create a safer and simpler VLP and test its functional efficacy to transiently degrade SAMHD1 and enhance Ara-C cytotoxicity in AML cell lines and primary AML blasts.

## Materials and methods

### Plasmids and cloning

The 2nd generation VLP backbone plasmid was generated by first inserting unique restriction sites, *BlpI* and *XhoI*, into the 1st generation pSIV3 + plasmid [18] using a 3-step standard PCR protocol with 5’ GATGCCCTACAGAATCAGAGAGCAG 3’ (forward) and 5’ CTCGAGGTGGCTAAGCAGTGAGCTATGCCACCTCTCTAG 3’ (reverse) for the first and 5’ GCTCACTGCTTAGCCACCTCGAGATGTACATTTATATTGGCTC 3’ (forward) and 5’ GTAACCATTATAAGCTGCAATAAACAAGTTAACAACAAC 3’ (reverse) for the second PCR reaction. This left the Rev-responsive element (RRE) intact, but the accessory proteins Vif, Vpx, Vpr, Tat, and Rev were removed. Subsequently, 3xFLAG-tagged Vpx/Vpr were inserted using the available *BlpI* and *XhoI* cutting sites [14].

SIVmac Vpx mutants were generated from the pSIV3 + 3xFLAG-VpxSIVmac239 construct by Gibson assembly cloning. PCR fragments containing the required amino acid substitutions were replaced using *BlpI* and *XhoI*. For the VpxSIVmac239 P64Q mutant, two fragments were generated using 5’-gagaggtggcatagctcactgc-3’ (forward) and 5’-gctcatgccctgctcgtcg-3’ (reverse), and 5’-gtaatgttggacatgagccaatataaatgtacatc -3’ (forward) and 5’-cacgacgagcagggcatgagccagagctacgtgaagtacagatac-3’ (reverse). For VpxSIVmac239 I75M, two fragments were generated using instead a different reverse primer for the second fragment: 5’- cacgacgagcagggcatgagccccagctacgtgaagtacagatacctgtgcctgatgcagaaggccctgttcatg-3’ (reverse). For the VpxSIVmac239 P64Q + I75M double mutant (equivalent to VpxSIVmac251), two fragments were generated with a new reverse primer for the second fragment: 5’- cacgacgagcagggcatgagccagagctacgtgaagtacagatacctgtgcctgatgcagaaggccctgttcatg -3’ (reverse). To generate pSIV3 + VpxSIVmac239 P64Q + I75M without the 3xFLAG-tag, an insert was generated by amplifying VpxSIVmac239 P64Q + I75M and adding overlapping sequences for Gibson assembly with the primers 5’-gagaggtggcatagctcactgc-3’ (forward) and 5’-gagaggtggcatagctcactgcttagccaccatgagcgaccccagagagagaatc-3’ (reverse). All generated plasmids were sequence verified by Sanger sequencing.

### Cell culture

HEK293T cells (DSMZ ACC635) were cultivated as described [19]. All AML suspension cell lines were cultivated in Iscove’s modified Dulbecco’s medium (IMDM, Sigma-Aldrich), supplemented with 10% fetal calf serum (FCS, Sigma-Aldrich), 100 units/mL penicillin, and 0.1 mg/mL streptomycin (Sigma-Aldrich). HEL SAMHD1 cells were generated by lentiviral transduction using pHR-SAMHD1 as transfer vector—successful transduction was monitored by intracellular SAMHD1 staining. The generation and cultivation of Ara-C resistant HEL (HEL Ara-C^r^) and HL-60 (HL-60 Ara-C^r^) cell lines have already been described [6]. The cells were maintained at a density of approximately 1 × 10^6^ cells/mL and incubated at 37 °C and 5% CO_2_.

### Patients

After written informed consent in accordance with the Declaration of Helsinki and approval by the Institutional Review Board of the Ludwig-Maximilians-University (Munich, Germany, reference number: 216-08), bone marrow (BM) samples were collected from patients with AML at primary diagnosis. Patient characteristics are summarized in Supplementary Table 1. Mononuclear cells from AML patients were isolated by density gradient centrifugation (Biochrom, Berlin, Germany) from BM samples and cryoconserved at below −80 °C in 80% FCS and 20% dimethyl sulfoxide (Serva Electrophoresis, Heidelberg, Germany). At diagnosis, a standard analysis of all samples was centrally done at the Laboratory for Leukemia Diagnostics, LMU University Hospital Munich. This included cytomorphology, cytogenetics, fluorescencein situ hybridization, and molecular genetics. For cytogenetic risk assessment refined MRC (medical research council) criteria were used. Combined cytogenetic and molecular risk stratification groups were assigned in accordance with the European LeukemiaNet (ELN) guidelines.

### Primary CD4 + T cell isolation and cultivation

The isolation and cultivation of primary CD4 + T cells from healthy donor blood were recently described [19]. The isolated cells were incubated at 37 °C and 5% CO_2_ in RPMI medium (Thermo Fisher Scientific) containing FCS and penicillin/streptomycin at a cell density of 2 × 10^6^ cells/mL without addition of cytokines.

### VLP production

For the production of 2nd generation VLPs, 26.25 µg of 2nd generation pSIV3 + plasmid encoding the appropriate Vpx/Vpr homolog, 3.55 µg of pCMV HIV-1 Rev plasmid, and 7.08 µg of the pMD2.G plasmid encoding the VSV-G envelope protein were used per 145 cm^2^ dish with 80–90% confluent HEK293T cells, unless otherwise stated. For the 1st generation VLPs, the Rev plasmid was replaced by an empty pcDNA plasmid, and the 1st generation pSIV3 + plasmid was used [6]. For the production of the BlaM-Vpr VLPs, HEK293T cells were transfected with 8.3 µg pCMV plasmid encoding BlaM-Vpr, 12.5 µg psPAX2 packaging vector, and 4.125 µg of pMD2.G plasmid. psPAX2 was a gift from Didier Trono (Addgene plasmid # 12,260; http://n2t.net/addgene:12260). 48–72 h post-transfection, the supernatant was filtered through 0.45 µm vacuum filters, overlaid onto 25% sucrose cushion, and subsequently subjected to ultracentrifugation at 110,000 × g for 2 h at 4 °C. After centrifugation, the supernatant was discarded, the VLPs were reconstituted in PBS for at least 30 min at 4 °C, thoroughly re-suspended, and stored at −80 °C.

### SG-PERT

The yield of VLPs was quantified via the SYBR Green I-based real-time PCR-enhanced reverse transcriptase (SG-PERT) assay [20]. Samples were run on the CFX96 BioRad qPCR machine, and results were analyzed using the CFX Maestro Software. The RT activity of the VLPs was determined as pRT units per µL.

### Primary CD4 + T cell nucleofection

Prior to subcloning, the efficacy of different FLAG-tagged Vpx/Vpr proteins [14] to degrade SAMDH1 in resting CD4 + T cells was tested. For this purpose, 625 ng FLAG-tagged Vpx/Vpr expression plasmids [14] together with 375 ng pMAX GFP were nucleofected into primary CD4 + T cells using the 4D-Nucleofector^®^ X Kit P3 (Lonza) according to the manufacturer’s recommendations (program EO-115).

### VLP titration

For VLP titration assays, 1 × 10^5^ THP-1 cells were distributed into each well of a flat-bottom 96-well plate in 200 µL of final volume. Different volumes of VLPs were added prior to spinoculation at 1200 × g for 90 min at 37 °C. The transduced cells were then incubated at 37 °C and 5% CO_2_ for 24 h before conducting SAMHD1 staining.

### VLP and Ara-C co-treatment

1 × 10^4^ cells per well of AML cell lines at 80 µL final volume were distributed into flat-bottom 96-well plates. 10 µL of VLPs were then added, and spinoculation was performed as described above. After 24 h, 10 µL of 1:4 serial dilutions of Ara-C were added to the VLP-treated cells and incubated for 96 h at 37 °C and 5% CO_2_. Subsequently, 10 µL of resazurin was added to each well and incubated for a further 5 h. The reduction of the blue dye resazurin to pink resorufin by viable cells was measured using the CLARIOstar Plus microplate reader at the wavelengths 600 nm and 570 nm, respectively. Resazurin reduction of untreated cells was used as control to calculate the percentage viable cells in all treated wells. All ex vivo cytotoxicity assays using AML blasts were performed as published previously [22]. The amount of viable primary AML blasts was determined by flow cytometry (Cytoflex S, Beckman Coulter, Brea, CA, USA). The following fluorochrome conjugated monoclonal antibodies were used (all with 1:50 dilutions): CD45 (HI30), CD33 (WM-53), CD34 (561) (all from BioLegend, San Diego, CA, USA). For dead cell discrimination, LIVE/DEAD^™^ Fixable Aqua Dead Cell Stain Kit (Thermo Fisher Scientific, Waltham, MA, USA) was used. Specific lysis was calculated as follows: % specific lysis = 100 –{number of viable CD33^+^ cells (Ara-C condition)/number of viable CD33^+^ cells (untreated control condition)} × 100.

### Intracellular staining of SAMHD1 and flow cytometry

The intracellular staining of SAMHD1 was conducted as previously described [19, 21]. Briefly, VLP-treated cells were washed with PBS and stained with Zombie Green/Zombie Violet^™^ fixable viability dye (Thermo Fisher Scientific, in DMSO, diluted 1:000 in PBS) for 15 min. Next, the cells were fixed using 4% paraformaldehyde, permeabilized with BD Phosflow Perm Buffer III, and stained with primary SAMHD1 antibody (Proteintech), followed by secondary anti-rabbit antibody conjugated to AlexaFluor 660 (Invitrogen). Intracellular SAMHD1 levels were measured using the BD FACSLyric instrument and analyzed using the FlowJo software.

For the SAMHD1 staining of primary AML blasts, the cells were first washed in PBS and transferred to 96-well v-bottom plates. The cells were then stained for surface markers using 1:20 dilutions of the following antibodies: anti-CD33-PE (BioLegend), anti-CD34-FITC (BioLegend), and anti-CD45-V450 (BD). The cells were incubated for 30 min at 4 °C in the dark and washed in FACS stain buffer (PBS, 1% FCS, 0.09% sodium azide, 2 mM EDTA) before being fixed, permeabilized, and stained for SAMHD1 as described above. SAMHD1 levels were determined in CD45 + AML blast cells using the BD FACSLyric instrument and analyzed using the FlowJo software.

For surface staining of primary CD4 + T cells, samples were treated analogously to primary blasts except that anti-hCD4-FITC (Invitrogen) diluted 1:100 in PBS was used instead.

### SDS-PAGE and western blot

Total protein from AML cells were extracted as recently described [19]. Briefly, cells were lysed in Hunt lysis buffer via freeze–thaw cycles in liquid nitrogen and centrifuged at top speed at 4 °C for 30 min. The protein-containing supernatant was transferred to a fresh tube. An appropriate volume of total protein was diluted in PBS. 4 × Laemmli buffer was added to the diluted total protein and boiled for 5 min at 95 °C. For SDS-PAGE analysis of VLPs, VLPs were prediluted in PBS before adding 4 × Laemmli buffer. 12% polyacrylamide gels were prepared according to manufacturer’s instructions (Thermo Fischer Scientific, SureCast system). PageRuler Plus Prestained protein ladder (Thermo Fisher Scientific) was used as a ladder. Electrophoresis was performed at constant voltage of 100 V for 1 h, followed by wet transfer of the separated proteins onto a nitrocellulose membrane at 10 V for 1 h. The membranes were blocked using 5% milk in TRIS-buffered saline with 0.1% Tween-20 for 30 min before performing immunoblotting with the appropriate antibodies. Rabbit anti-SAMHD1 (Proteintech, 1:1000), mouse anti-vinculin (Sigma-Aldrich, 1:2000), anti-p27 (hybridoma supernatant, 1:100), and anti-FLAG (Sigma-Aldrich, 1:1000) were used as primary antibodies to detect the respective proteins. Species-specific antibodies conjugated with horseradish peroxidase at 1:10,000 dilution were used as secondary antibody, followed by staining using either the Clarity^™^ Western ECL Kit (BioRad) or the SuperSignal^™^ West Femto Maximum Sensitivity Substrate kit (Thermo Fisher Scientific). Membranes were imaged on the Vilber Fusion FX machine.

### Automated SDS-PAGE and western blot using protein simple jess system

For the detection of SAMHD1 from low amounts of total protein, the Jess automated SDS-PAGE and western blot system was used as per manufacturer’s instructions. Mouse anti-SAMHD1 (1:200 dilution, customized, kindly provided by Dr. Keppler) and mouse anti-vinculin (1:2000 dilution, Sigma-Aldrich) antibodies were used with the anti-mouse detection module provided by ProteinSimple. For the separation of the proteins, a 13-well 12–230 kDa separation module was chosen.

### Virion fusion assay

In order to determine the VSV-G-dependent fusion efficiency, a virion fusion assay was performed [23]. First, 2 × 10^5^ AML cell lines or primary AML blasts were seeded in U-bottom 96-well plates. 1 × 10^10^ pRTU/µL of BlaM-Vpr VLPs were then added to the cells and spinoculated as described above. The cells were incubated for a further 2.5 h at 37 °C and 5% CO_2_, pelleted, and the supernatant was aspirated. Cells were stained with CCF4 staining solution (Thermo Fisher Scientific). 1 mL of the staining solution contained 2 µL of the CCF4 dye, 8 µL Solution B (LiveBLAzer^™^ FRET-B/G Loading Kit, Thermo Fisher Scientific), and 10 µL Probenecid, diluted in CO_2_-independent medium (Thermo Fisher Scientific) at room temperature in the dark overnight, wrapped in wet tissue to avoid evaporation. The following day, the cells were washed, fixed with 4% paraformaldehyde, and subjected to flow cytometry. A shift in the emission from 520 to 450 nm represents the cleavage of the CCF4 by the BlaM-Vpr fusion protein, indicating successful fusion of the VLP with the target cell.

### Ara-CTP measurement

The concentrations of ^13^C_3_-Ara-CTP in the samples treated with VLPs were analyzed by liquid chromatography–electrospray ionization-tandem mass spectrometry (LC-MS/MS) essentially as previously described [6]. In brief, 2.5 × 10^5^ THP-1 cells in a 12-well format with 1 ml culture medium/well were treated with 25 µl of purified and concentrated VLPs by centrifugation at 1200 × g for 90 min at 37 °C. After 20 h, the cells were treated with 10 µM ^13^C_3_-Ara-C for 6 h, harvested, washed once with PBS, and subjected to LC-MS/MS measurement.

### In silico structural predictions

Structural predictions of SIV mac239 Vpx, SIV mac239 VpxP64Q, SIV mac239 VpxI75M, and the double mutant equivalent to SIV mac251 Vpx were generated using AlphaFold [24, 25]. Here, the user interface ChimeraX was used [26, 27] with the preinstalled AlphaFold structure prediction tool using ColabFold software coupled to Google Colaboratory [28].

### Statistical analysis

Statistical analyses were carried out with Prism 9 (GraphPad). Data are visualized as the mean and the standard error of the mean (S.E.M). See figure legends for the statistical test applied in each case. Cell viability curves were modeled using a nonlinear fit in Prism 9 (Absolute IC_50_, X is concentration, baseline constraint set to 0).

## Results

### Characterization of minimized SIV-based VLPs

**Fig. 1:**
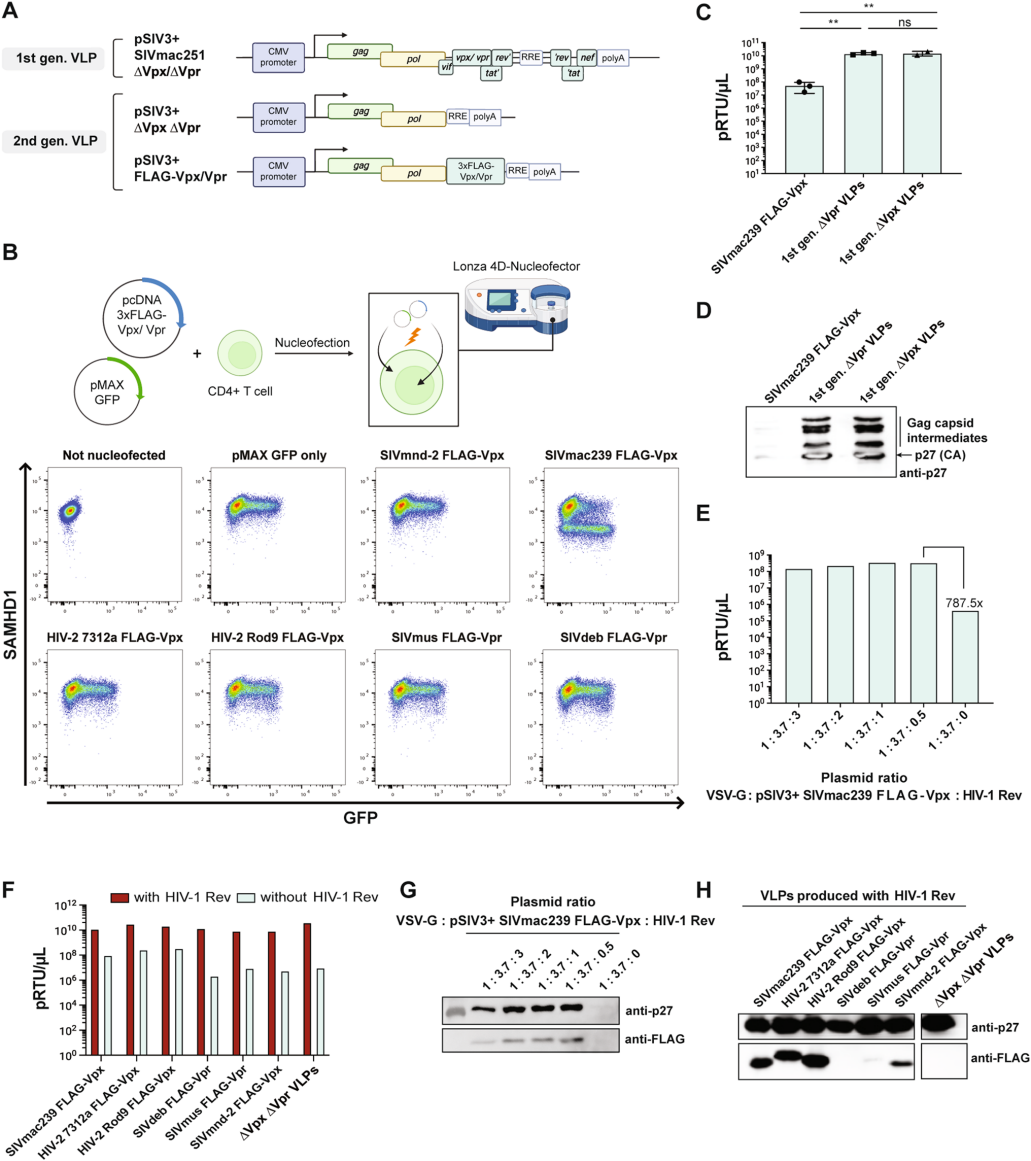
Successful generation, characterization and optimization of 2nd generation VLPs. **A** depicted is the schematic representation of 1st and 2nd generation VLPs. **B** depicted is a schematic and representative primary dot blots of resting CD4 + T cells transfected with Vpx/Vpr expression plasmids together with pMAX GFP. Intracellular SAMHD1 levels were analyzed 16 h post-transfection by flow cytometry. **C** Representative quantification of RT activity by SG-PERT after VLP purification showing mean ± S.D. with technical replicates. **D** representative Western Blot analysis for p27 capsid levels after VLP purification. **E** RT activity measurement of VSV-G-pseudotyped SIV3 + 3xFLAG-SIVmac239 Vpx particles after titration of HIV-Rev during VLP production. **F** RT activity measurement for a panel of 2nd generation VLPs in the presence (red) or absence (gray) of HIV-Rev using the ratio 1:3.7:0.5 during VLP production. **G** representative Western Blot analysis for p27 capsid levels after VLP purification in the presence or absence of HIV-Rev. **H** representative Western Blot analysis of purified 2nd generation VLPs for p27 capsid and FLAG as a surrogate for Vpx/Vpr expression. Statistical analyses were performed using one-way ANOVA. **p < 0.01, n.s. not significant

We and others previously established a negative correlation of SAMHD1 expression and Ara-C cytotoxicity [6, 7]. Based on our previous observations that Vpx-containing VLPs transiently degrade SAMHD1 and thus ameliorate Ara-C cytotoxicity in vitro and in vivo [6], we designed a minimized 2nd generation VLP devoid of accessory and regulatory proteins, especially omitting Vpx and Vpr (Fig. 1A). Since primary resting CD4 + T cells express equivalent levels of SAMHD1 compared to primary myeloid cells, but are easier to manipulate (data not shown), we decided to use these primary cells to select which Vpx/Vpr should be incorporated into the 2nd generation VLP. We nucleofected primary resting CD4 + T cells with 3xFLAG-tagged Vpx/Vpr expression plasmids together with pMAX GFP and stained for intracellular SAMHD1 levels (Fig. 1B; Supplementary Fig. 1). pMAX GFP was used as a surrogate for co-transfected cells to better visualize SAMHD1 degradation. As expected from our previous analyses [21, 29], SIVmac239 Vpx was able to degrade SAMHD1 in contrast to SIVmnd-2 Vpx. Interestingly, the effect of HIV-2 7312A Vpx and HIV-2 Rod9 Vpx was less pronounced despite our previous results [21]. In addition, SIVdeb Vpr and SIVmus Vpr were able to induce a slight SAMHD1 degradation comparable to the HIV-2 Vpx, in agreement with previous findings [14]. All Vpx/Vpr were then subcloned into the 2nd generation VLPs, preserving almost the natural localization of Vpx/Vpr and thus LTR-driven expression and complex splicing (Fig. 1A). The VLPs were purified and their reverse transcriptase (RT) quantity was measured by SG-PERT (Fig. 1C). An ~ 270-fold difference in RT activity was observed between the 1st and 2nd generation VLPs. This was also consistent with the different levels of structural polyprotein Gag and p27 capsid detected by Western blotting (Fig. 1D). Rev mediates the nuclear export of unspliced SIV transcripts by binding to the Rev-responsive element (RRE) [30]. Since the 2nd generation VLPs retained their RRE, we reasoned that the addition of Rev might increase the VLP yield. As assumed, the addition of minute amounts of HIV-1 Rev (ratio: 1 × VSV-G: 3.7 × SIV3 + SIVmac239 Vpx: 0.5 × HIV-1 Rev) increased VLP yields and p27 capsid levels to 1st generation levels (Fig. 1E, G) and this was independent of the Vpx/Vpr insertion (Fig. 1F). The incorporation of the different 3xFLAG-tagged Vpx/Vpr was analyzed by Western blotting (Fig. 1H). Since there is no good antibody available to detect the expression of Vpx/Vpr, the FLAG tag was used as a surrogate to measure the incorporation of Vpx/Vpr into the 2nd generation VLPs, unfortunately this was not possible for the 1st generation VLPs. Differences in packaging efficiency were observed, especially for the Vpr-expressing VLPs, and only VLPs with good to very good incorporation levels were subsequently used. In conclusion, minimized SIV-based VLPs were successfully generated and optimized for Vpx protein delivery.

### Functional efficacy of 2nd generation VLPs in AML cell lines

**Fig. 2:**
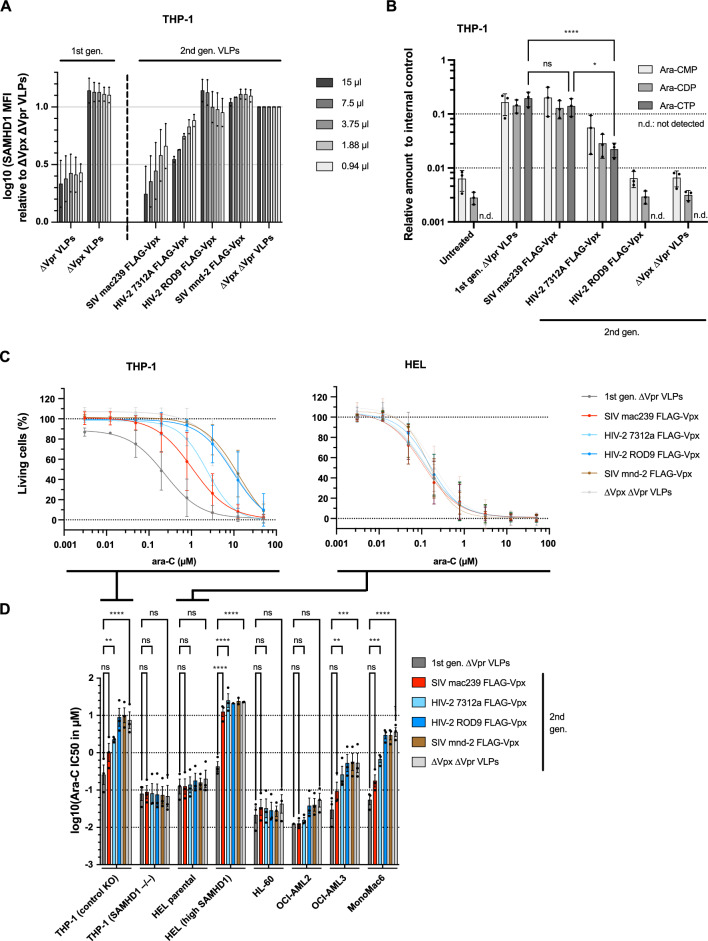
2nd generation SIVmac239 Vpx and HIV-2 7312a Vpx efficiently degrade SAMHD1 and enhance Ara-C cytotoxicity. **A** THP-1 cells were transduced with VSV-G-pseudotyped 1st and 2nd generation VLPs. 24 h post-transduction, intracellular SAMHD1 levels were analyzed by flow cytometry. Depicted is a representative titration experiment with means ± S.E.M of technical replicates. **B** THP-1 cells were transduced with VSV-G-pseudotyped 1st and 2nd generation VLPs. 24 h post-transduction, cells were treated with ^13^C_3_-Ara-CTP and Ara-CMP, Ara-CDP and Ara-CTP levels were quantified by LC-MS/MS. Shown are means ± S.E.M. of three biological replicates. **C** THP-1 and HEL cells were transduced with VSV-G-pseudotyped 1st and 2nd generation VLPs. 24 h post-transduction, cells were treated with different Ara-C concentrations and the percentage of living cells was analyzed using the Resazurin assay. Shown is a representative titration curve analysis. **D** AML cell lines were essentially treated as described for (**C**). Depicted are means ± S.E.M. of 2–3 independent experiments. Statistical analyses were performed using two-way ANOVA with Dunnett’s multiple comparisons test with a single pooled variance. The 1st generation VLPs were used as reference. **p < 0.01, ***p < 0.001, ****p < 0.0001, n.s. not significant

Next, we sought to test the SAMHD1 degradation capacity of the 2nd generation VLPs. THP-1 cells, which naturally express high levels of endogenous SAMHD1, were transduced with different amounts of 1st and 2nd generation VLPs (Fig. 2A). In contrast to the nucleofection experiment (Fig. 1B) and despite very good incorporation into VLPs (Fig. 1H), both SIVmac239 Vpx and HIV-2 7312a Vpx were able to degrade SAMHD1 as 1st generation Vpx-containing VLPs (ΔVpr VLPs), with only partial SAMHD1 degradation at lower titration volumes (Fig. 2A). HIV-2 ROD9 Vpx and both Vprs were unable to induce SAMHD1 degradation in the VLP context. This was also confirmed by quantitative Western blotting (Supplementary Fig. 1). Vpx-mediated SAMHD1 degradation should lead to increased levels of Ara-CTP, the substrate recognized by SAMHD1. Here, no significant difference in Ara-CTP levels was observed between the 1st generation VLP and the 2nd generation SIVmac239 Vpx VLP (Fig. 2B). In comparison, Ara-CTP levels were approximately ninefold lower with HIV-2 7312a Vpx and undetectable with HIV-2 Rod9 Vpx and the empty 2nd generation control (ΔVpx ΔVpr VLP). Next, different AML cell lines that either naturally express high (THP-1, OCI-AML2, OCI-AML3, MonoMac6) or low (HEL, HL-60) levels of SAMHD1, stably overexpress SAMHD1 (HEL (high SAMHD1)), or in which SAMHD1 has been knocked out (THP-1 (SAMHD1 -/-)) were used (Supplementary Fig. 2). The percentage of viable cells was then measured after the addition of different concentrations of Ara-C and the IC50 values were calculated (Fig. 2C, D). Of note, treatment with the 1st generation VLPs in the absence of Ara-C slightly induced cytotoxicity in THP-1 cells compared to our 2nd generation VLPs (Fig. 2C, first data point), suggesting additional cytotoxic properties lacking in our 2nd generation VLPs.

As expected, VLP treatment had no effect on Ara-C cytotoxicity in the SAMHD1 low cells. Interestingly, no statistically significant difference was observed between 1st generation VLP and 2nd generation 3xFLAG-tagged SIVmac239 Vpx VLP in SAMHD1 high cells, only in HEL cells stably overexpressing SAMHD1 (dark gray bar vs. red bar, Fig. 2D). Consistent with previous results, HIV-2 7312a was less efficient than SIVmac239 Vpx, and HIV-2 Rod9 was unable to enhance Ara-C cytotoxicity, comparable to SIVmnd-2 Vpx and the empty control (ΔVpx ΔVpr VLP).

### Investigating the difference between 1st and 2nd generation VLPs

**Fig. 3:**
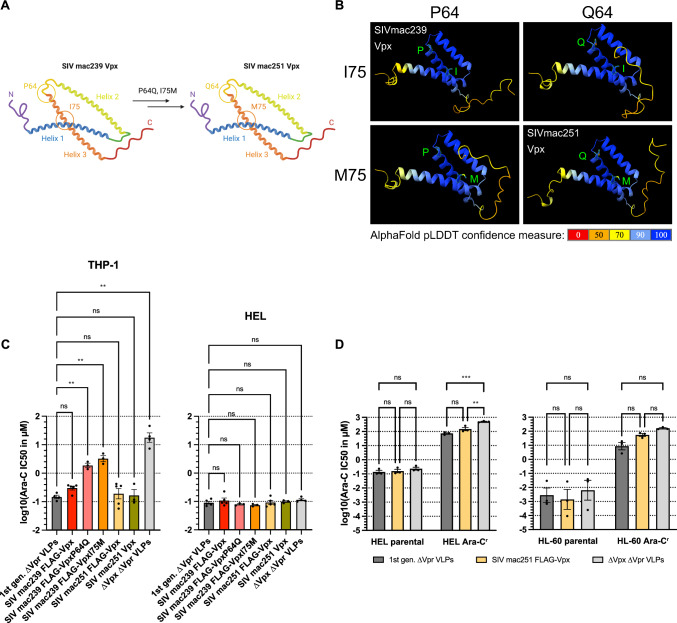
Single amino acid substitutions reduce the efficacy of Vpx to enhance Ara-C cytotoxicity. **A, B** depicted is a schematic (**A**) and AlphaFold prediction (**B**) for SIV mac239 Vpx, mutants thereof, and SIVmac251 Vpx. Modeling was done using ChimeraX with AlphaFold. **C,** THP-1 and HEL cells were essentially treated as described for figure legend **2C**. Depicted are means ± S.E.M. of 3 to 5 independent experiments. Statistical analyses were performed using two-way ANOVA with Dunnett’s T3 multiple comparisons test with individual variances computed for each comparison. **D** parental and Ara-C^r^ HEL and HL-60 cells were essentially treated as described for figure legend **2C**. Depicted are means ± S.E.M. of 3 independent experiments. Statistical analyses were performed using two-way ANOVA with Dunnett’s T3 multiple comparisons test with individual variances computed for each comparison. The 1st generation VLPs were used as reference. **p < 0.01, ****p < 0.0001, n.s. not significant

Since the 2nd generation 3xFLAG-tagged SIVmac239 Vpx VLPs were closest to the 1st generation VLPs in terms of SAMHD1 degradation and increase in Ara-C cytotoxicity, but slight tendencies for better performance of the 1st generation VLPs were observed (Fig. 2D), we sought to investigate the difference between the two. The 1st generation VLP encodes SIVmac251. The two Vpx differ by only two amino acids (SIVmac239 P64 and I75, SIVmac251 Q64 and M75) and the 3xFLAG-tag (Fig. 3A, B). Analyzing the Ara-C IC50 values with different Vpx mutants based on the 2nd generation 3xFLAG-tagged SIVmac239 Vpx, we found no differences in HEL cells, which have naturally low SAMHD1 expression. In THP-1 cells, treatment with the single mutants P64Q and I75M led to a 6- and 11-fold diminished effect on improving Ara-C cytotoxicity, respectively, compared to 3xFLAG-SIVmac239 Vpx. When both amino acids were replaced to match either SIVmac239 or SIVmac251 Vpx, the effect on improving Ara-C cytotoxicity was restored. In addition, the 3xFLAG-tag did not appear to affect performance (Fig. 3C). We also performed similar experiments with Ara-C^r^ cell lines [6] to observe whether cell lines that have acquired resistance to Ara-C behave differently when treated with 1st and 2nd generation VLPs. Both 1st and 2nd generation VLPs were able to sensitize Ara-C resistant HL-60 cells to Ara-C to a similar extent as before [6]. A similar trend, although not statistically significant, was also observed for the Ara-C resistant HEL cells. Again, we did not observe statistically significant differences between 1st and 2nd generation VLPs, indicating that simplification of the VLP does not drastically alter its performance.

### Functional efficacy of 2nd generation VLPs in primary AML blasts

**Fig. 4:**
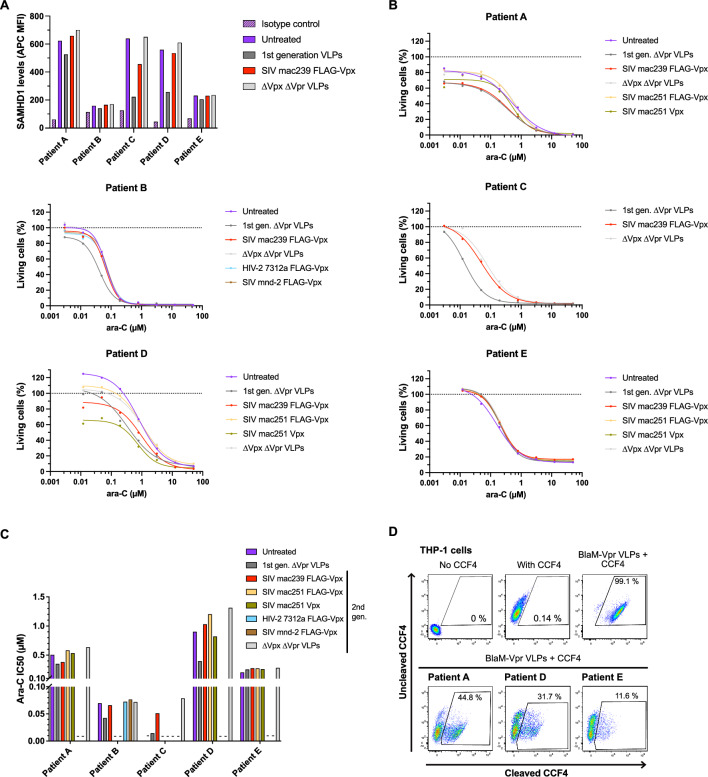
Primary AML cells are less responsive to VSV-G-pseudotyped 1st and 2nd generation VLPs. **A** Patient BM samples were transduced with VSV-G-pseudotyped 1st and 2nd generation VLPs. 24 h post-transduction, intracellular SAMHD1 levels were analyzed by flow cytometry. **B** Patient BM samples were transduced with VSV-G-pseudotyped 1st and 2nd generation VLPs. 24 h post-transduction, cells were treated with Ara-C and the percentage of living cells was analyzed by flow cytometry. **C** Ara-C IC_50_ values were calculated based on (**B**). **D** Patient BM samples were transduced with Blam-Vpr VLPs and transduction efficiency was analyzed as percentage of cleaved CCF4-AM substrate by flow cytometry

In addition to AML cell lines, we tested our 2nd generation VLPs on primary AML blasts derived from treatment-naïve, initially diagnosed patients (Fig. 4, Supplementary Table 1). Out of five patients, only patients C and D showed reasonable SAMHD1 degradation levels even with the 1st generation VLPs, and the 2nd generation SIVmac239 Vpx VLPs only had an effect in patient C with an approximately twofold lower efficiency (Fig. 4A, Supplementary Fig. 3). This was also reflected in the Ara-C IC50 determinations (Fig. 4B, C), where on average approximately threefold improvements were observed. We reasoned that VSV-G-driven entry of VLPs might explain the difference between AML cell lines and primary AML blasts. Therefore, we performed a virion fusion assay [23] (Supplementary Fig. 4A) and measured only 12% to 45% virion entry in primary AML blasts (Fig. 4D), in contrast to up to 96% entry in AML cell lines (Supplementary Fig. 4B), explaining the lower efficacy in primary AML blasts. We also performed a TCGA database analysis of LDLR mRNA expression in AML patients and compared this with CD33, SAMHD1, and PSA (prostate specific antigen), the latter serving as a negative control (Supplementary Fig. 5C). Taken together with data from the Human Protein Atlas (Cell line—LDLR—The Human Protein Atlas), LDLR mRNA expression appeared to be ubiquitous at varying levels, suggesting that functional protein expression may differ between cell lines and primary cells. Notably, similar to our observation with THP-1 cells (Fig. 2C), we observed a mild cytotoxicity of the 1st generation VLPs, especially in patients B and C (Fig. 4B).

In conclusion, 1st and 2nd generation VLPs have reduced efficacy in primary AML blasts, which may be explained by lower transducibility using VSV-G-pseudotyped particles.

## Discussion

VLPs based on lentiviruses such as HIV or SIV are devoid of the envelope but contain all accessory and regulatory proteins. While these proteins perform a primary function necessary for viral replication, secondary functions have been described that alter host cell networks and induce a variety of cellular responses [31]. In order to get rid of these most likely unwanted effects, our intention was to create a simpler and thus safer VLP for Vpx delivery into AML cells (Fig. 1A). Indeed, we observed mild cytotoxic trends for the 1st generation VLPs in cell lines and primary cells in at the absence very low levels of Ara-C (Fig. 2C, Fig. 4B). To ensure that the primary function of Vpx to degrade SAMHD1 was retained, we performed a proof-of-concept study. Since our minimized VLPs were not codon-optimized, they retained their Rev dependence, meaning that Rev interacted with the RRE to export unspliced or incompletely sliced mRNA transcripts to the cytoplasm. Thus, in the absence of Rev, a reduction in VLP production was observed (Fig. 1C, D). As shown by others [32], only minute amounts of Rev were required to overcome this first hurdle (Fig. 1E, G).

Vpx/Vpr proteins are incorporated into VLPs through interaction with the p6 domain within the structural polyprotein Gag [33]. Apparently, the p6 domain of the VLP backbone derived from SIVmac251 was inefficient in incorporating Vpr proteins derived from SIVdeb and SIVmus (Fig. 1H). While the levels of Vpx incorporation were relatively comparable (Fig. 1H), it was striking that HIV-2 Rod9 Vpx was unable to degrade SAMHD1 (Fig. 2A) or increase Ara-CTP levels (Fig. 2B). This was in contrast to our transfection approach (Fig. 1B) and to a previous study [21], implying that the successful incorporation of HIV-2 Rod9 Vpx into SIV-based VLPs via the p6 domain somehow inactivated its natural function to degrade SAMHD1. Unfortunately, due to dilution effects, it was not possible to follow the fate of HIV-2 Rod9 Vpx in the cell after delivery of FLAG-tagged Vpx. Thus, in order to investigate the potency of SIVdeb/SIVmus Vpr as well as HIV-2 Rod9 Vpx in the future, p6 domain-independent incorporation mechanisms would have to be introduced.

Among the remaining Vpx orthologs, SIVmac239 Vpx outperformed HIV-2 7312A Vpx in degrading SAMHD1 (Fig. 2A), restoring Ara-CTP levels (Fig. 2B), and increasing Ara-C cytotoxicity (Fig. 2C, D), while SIVmnd-2 Vpx remained inactive, consistent with previous reports [29]. Interestingly, no significant difference between 1st and 2nd generation VLPs was observed in AML cells that naturally express high levels of SAMHD1. Stably overexpressed SAMHD1 in HEL cells was expressed from the CMV promoter. Therefore, we speculated that this may have an impact on the degradation susceptibility between 1st and 2nd generation VLPs.

Compared to the 1st generation, we saw only small nonsignificant trends that the 2nd generation VLPs were not as potent as the 1st generation VLPs. Therefore, we compared SIVmac239 and SIVmac251 Vpx at the amino acid level (Fig. 3A, B) and found only two amino acids that differed between them, next to the FLAG tag that we introduced in the 2nd generation VLPs for detection purposes. Here, we saw that the FLAG tag had no effect on the efficacy of Vpx to degrade SAMHD1 and thus enhance Ara-C cytotoxicity (Fig. 3C). Interestingly, both viruses are derived from two individual macaques. Although they are highly similar at the genomic level, differentin vivo properties have been described [34, 35]. Not having the optimal amino acid sequence of one or the other could lead to steric hindrance or subtle conformational changes of Vpx to target SAMHD1 to the DCAF1 proteasome complex and thus explain why the exchange of one amino acid reduced the efficacy up to 11-fold. In particular, amino acid 75 is close to the known Q76 position, which is essential for Vpx binding to DCAF1 [12]. Furthermore, amino acid 75 is close to the H78 position, which is important for the zinc-binding motif identified by Schwefel *et al*. [36]. Thus, subtle changes in the tertiary structure, as shown in Fig. 3D and in agreement with the resolved crystal structure [36], could also affect zinc binding. On the other hand, amino acid 64 is close to the di-tyrosine motif and R66 [36], which are important for SAMHD1 binding and part of the network connecting Vpx to DCAF1, respectively.

Our results suggest that both amino acids at positions 64 and 75 must be derived from either SIVmac251 or SIVmac239 to maintain full functionality. Unfortunately, due to the lack of a good available Vpx antibody, we were unable to determine how much Vpx protein is actually incorporated into the VLPs as such, whether this might explain the subtle differences between SIVmac251 Vpx (1st generation) versus 3xFLAG-tagged SIVmac239 Vpx (2nd generation). Interestingly, both 1st and 2nd generation VLPs were able to sensitize the Ara-C resistant HEL cell line, less so for HL-60. The effect was less pronounced than in the non-Ara-C resistant cell lines, but in the same range as previously shown [6]. This suggests that simplification of the VLP does not drastically alter its efficacy, but may help to reduce its immunogenicity/cytotoxicity (Fig. 2C, Fig. 4B) or other downstream problems due to the expression of regulatory and accessory proteins.

Application of both 1st and 2nd generation VLPs to primary AML blasts showed that the efficacy to degrade SAMHD1 and increase Ara-C cytotoxicity was low compared to cell lines (Fig. 2) and varied from donor to donor (Fig. 4). One reason may be the low susceptibility to VSV-G (Supplementary Fig. 3). More recently, differential expression of LDLR receptors, the target receptor for VSV-G, has been reported [37]. Analysis of the TCGA database of AML patients and comparison of these results with the Human Protein Atlas indicated that LDLR mRNA levels are ubiquitously expressed, although levels may vary. However, the functionality addressed by the virion fusion assays suggested that protein expression/function appears to differ between cell lines and primary AML blasts. To address this issue, future research should be conducted to improve the targeting of primary AML blasts. Indeed, a more targeted delivery using an engineered fusogen combined with a specific receptor or ligand has recently been published [38]. Due to the heterogeneity of AML, specific targeting has been and continues to be a challenge [39]. However, recent studies have suggested that co-expression of receptors such as CD33/TIM3 [40, 41] may lead to preferential targeting of AML blasts. This targeting approach could improve transduction efficiency, making it superior to other delivery methods such as nanoparticles or non-viral methods, and would not have problems with endosomal entrapment such as nano-metallo-organic frameworks (nanoMOFs). Even though small molecule inhibitors against SAMHD1 might hold promise as an alternative approach, current approaches still face many challenges such as drug resistance development and low response rate [42].

In conclusion, our study has succeeded in minimizing VLPs to deliver Vpx to AML cells, which would reduce any adverse effects derived from the accessory and regulatory proteins. A future bottleneck is to increase the transduction efficiency of primary AML blasts by combining different receptors described in the literature to increase the targeting of AML cells.

## Supplementary Information

Below is the link to the electronic supplementary material.

Supplementary file1 (PDF 252 KB)

Supplementary file2 (PDF 149 KB)

Supplementary file3 (PDF 333 KB)

Supplementary file4 (PDF 850 KB)

Supplementary file5 (PDF 550 KB)

Supplementary file6 (PDF 119 KB)

## Data Availability

The datasets generated during and/or analyzed during the current study are available from the corresponding author on reasonable request.
