# Supplementary material for: Novel Vpx virus-like particles to improve cytarabine treatment response against acute myeloid leukemia
Source: Clin Exp Med. 2024 Jul 13;24(1):155. doi: 10.1007/s10238-024-01425-w (PMC11246277; doi:10.1007/s10238-024-01425-w)
Supplement: Supplementary file 1 — Supplementary file1 (PDF 252 KB) [file 10238_2024_1425_MOESM1_ESM.pdf]

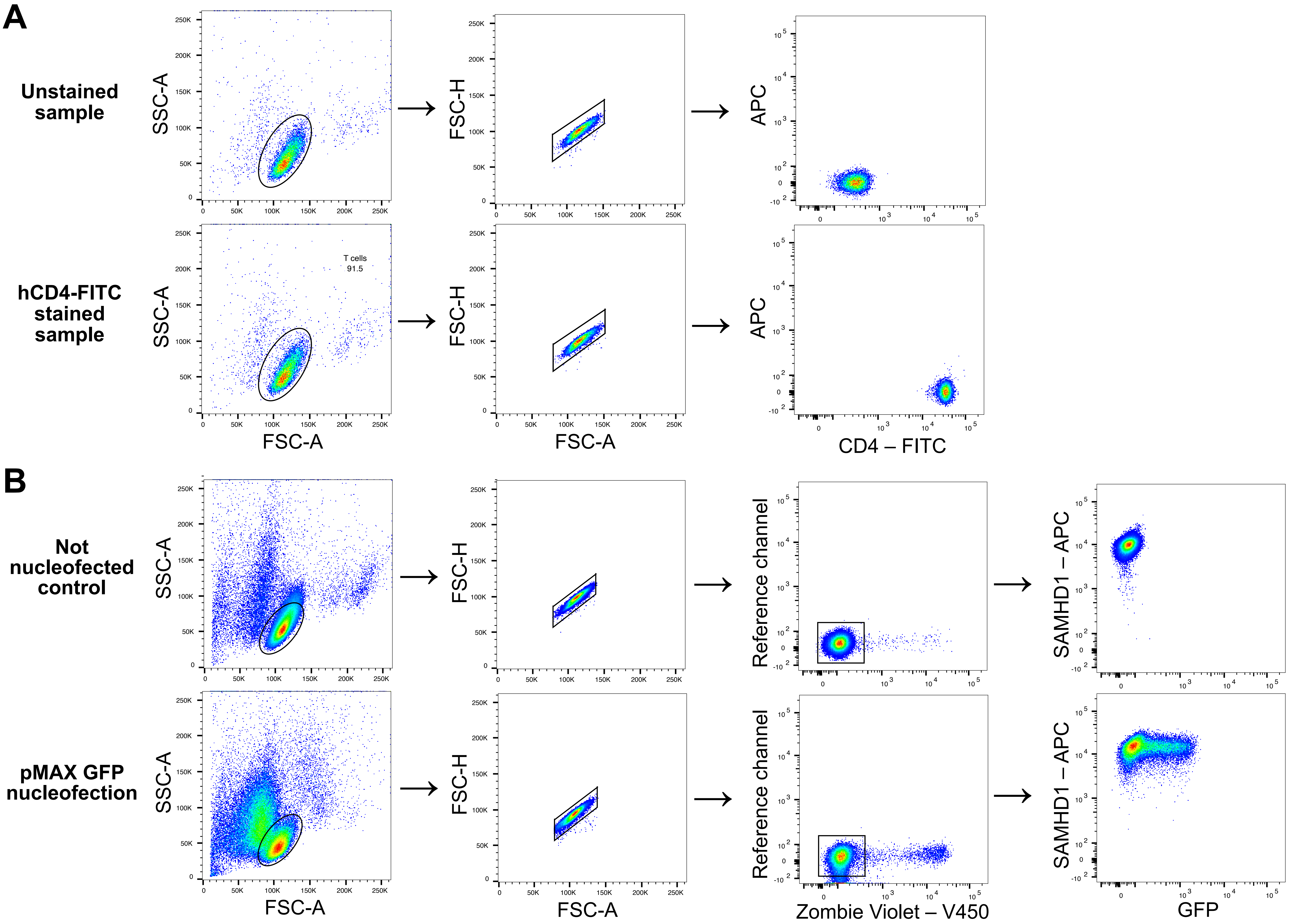

Supplementary Figure 1: Gating strategy for primary resting CD4 + T cells. Primary resting CD4 + T cells were isolated by negative selection according to the manufacturer's protocol. **A,** Shown are representative dot blots for freshly isolated CD4 + T cells that were stained for CD4 or left unstained and analyzed by flow cytometry. Cells were first gated using the forward scatter (FSC) and side scatter (SSC). Next, single cells were gated using the height (FSC-H) and area (FSC-A) of the forward scatter. From these, CD4 staining was analyzed. **B,** primary resting CD4 + T cells were nucleofected. Representative dot blots 20h post nucleofection are shown. In addition to the basic gating strategy as described in **A**, nucleofected cells were stained with Zombie Violet to distinguish living from dead cells. From these, SAMHD1 expression level was analyzed in addition to GFP.
