# Supplementary material for: Novel Vpx virus-like particles to improve cytarabine treatment response against acute myeloid leukemia
Source: Clin Exp Med. 2024 Jul 13;24(1):155. doi: 10.1007/s10238-024-01425-w (PMC11246277; doi:10.1007/s10238-024-01425-w)
Supplement: Supplementary file 2 — Supplementary file2 (PDF 149 KB) [file 10238_2024_1425_MOESM2_ESM.pdf]

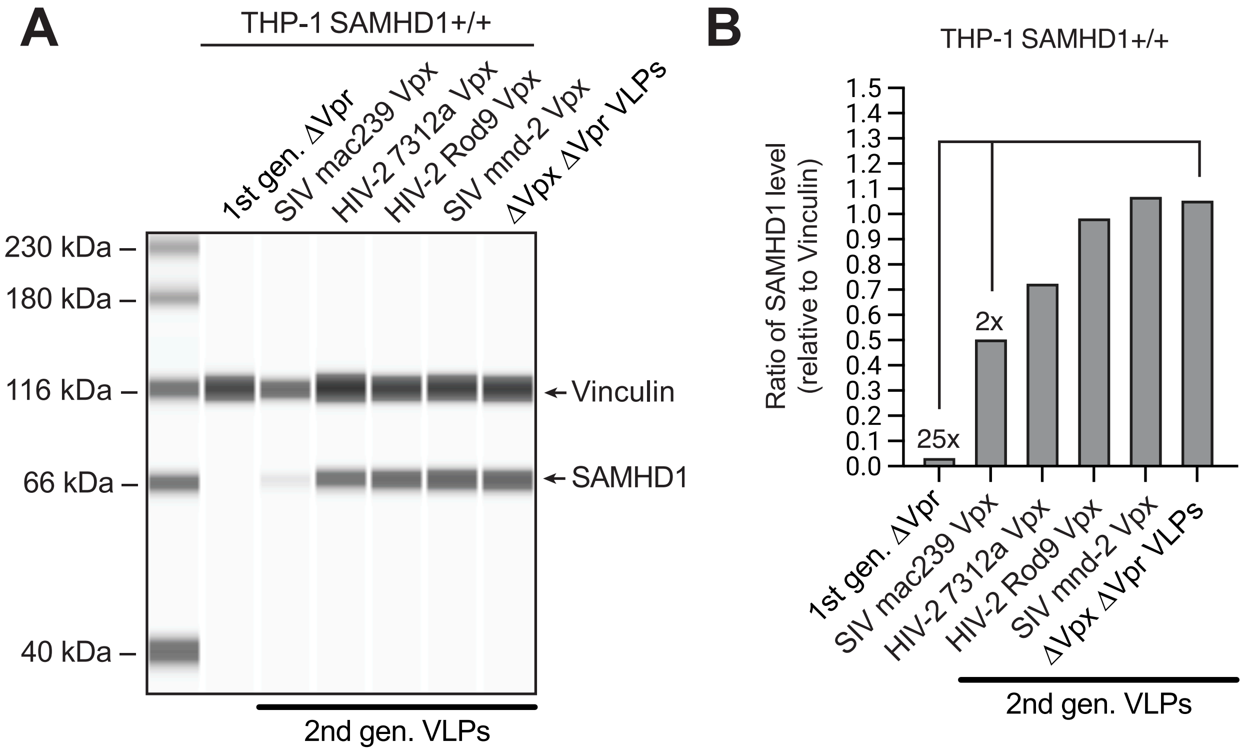

Supplementary Figure 2: SAMHD1 degradation efficacy varies between different Vpx alleles. THP-1 cells were essentially treated as described in the legend of Figure 2. 24 h post-transduction, cells were harvested for quantitative automated Western blotting. **A,** Shown is a representative automated Western blot showing the expression levels of SAMHD1 and Vinculin. The latter was used as a loading control. **B,** the quantification from **A** is shown.
