# Supplementary material for: Novel Vpx virus-like particles to improve cytarabine treatment response against acute myeloid leukemia
Source: Clin Exp Med. 2024 Jul 13;24(1):155. doi: 10.1007/s10238-024-01425-w (PMC11246277; doi:10.1007/s10238-024-01425-w)
Supplement: Supplementary file 3 — Supplementary file3 (PDF 333 KB) [file 10238_2024_1425_MOESM3_ESM.pdf]

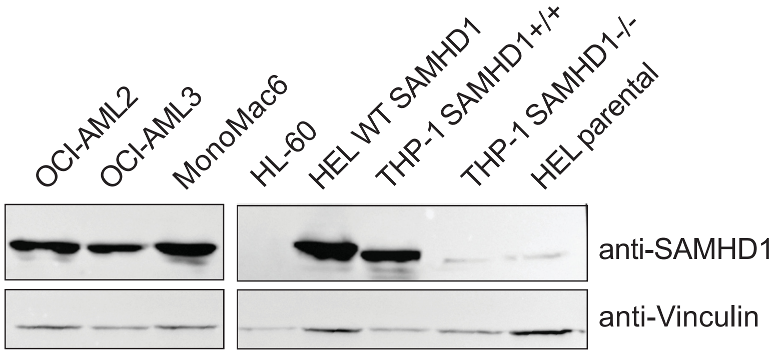

Supplementary Figure 3: Endogenous SAMHD1 expression levels vary among AML cell lines. Shown is a representative Western blot for SAMHD1 and Vinculin expression levels of the indicated cell lines. Vinculin was used as a loading control.
