# Supplementary material for: Novel Vpx virus-like particles to improve cytarabine treatment response against acute myeloid leukemia
Source: Clin Exp Med. 2024 Jul 13;24(1):155. doi: 10.1007/s10238-024-01425-w (PMC11246277; doi:10.1007/s10238-024-01425-w)
Supplement: Supplementary file 4 — Supplementary file4 (PDF 850 KB) [file 10238_2024_1425_MOESM4_ESM.pdf]

A

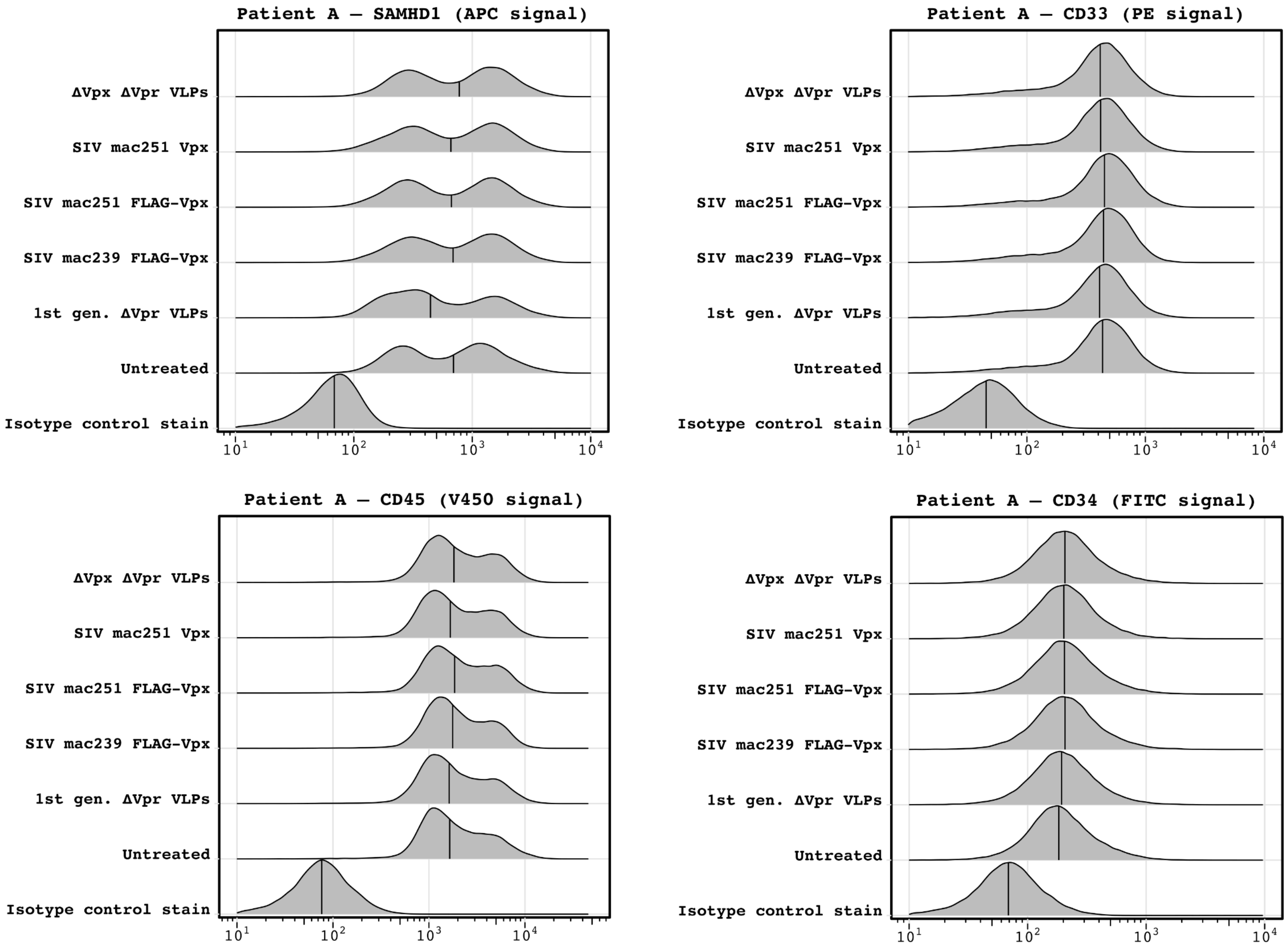

B

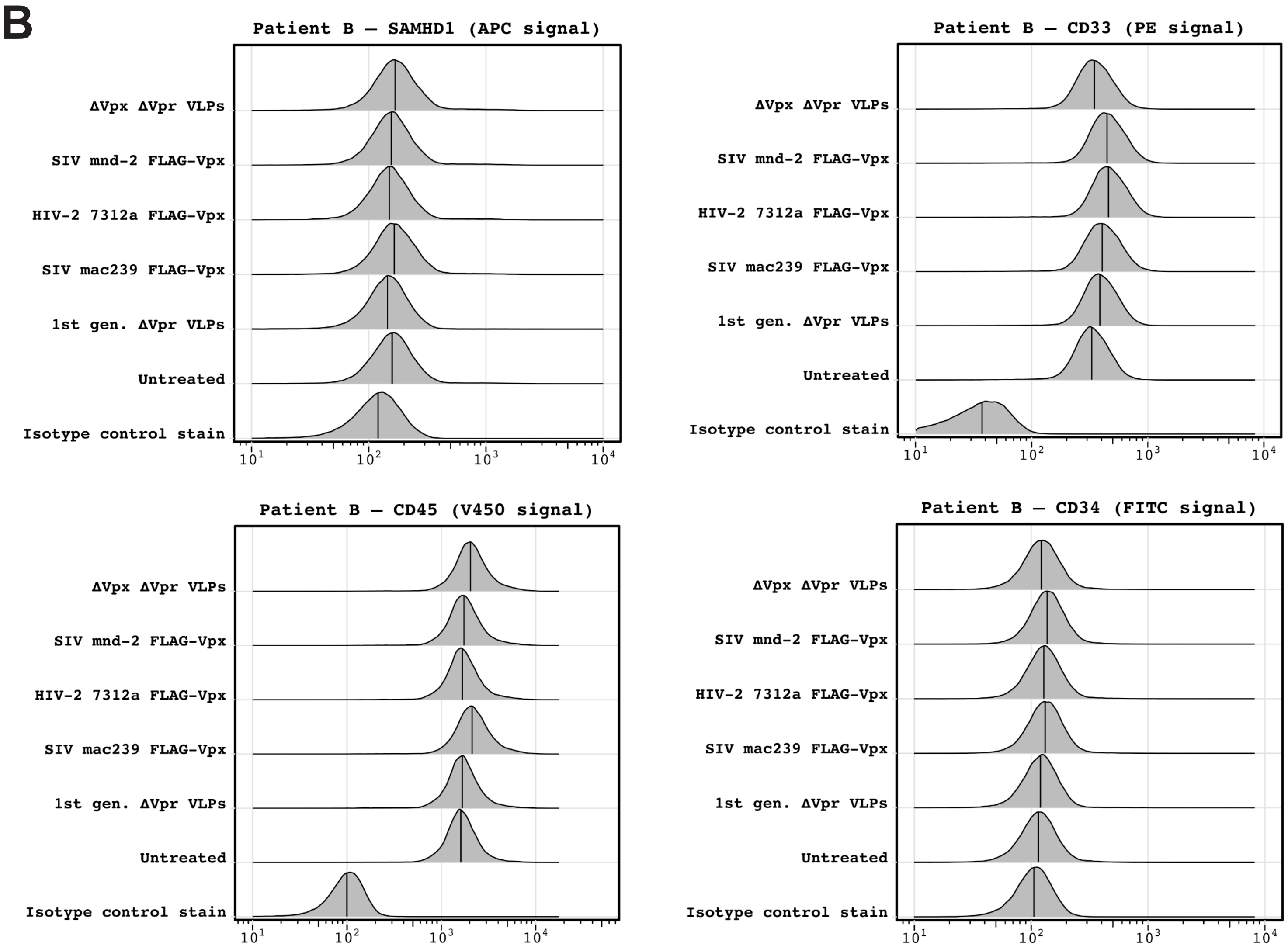

Supplementary Figure 4: Primary AML blasts respond poorly to VLP treatment in general. A, B, Primary AML blasts were treated as described in the legend of Figure 4. VLP-transduced samples were stained for SAMHD1, CD33, CD45 and CD34 and analyzed by flow cytometry. Shown are raw data for flow cytometric analyses derived from two patients shown in Figure 4A.
