# Supplementary material for: Novel Vpx virus-like particles to improve cytarabine treatment response against acute myeloid leukemia
Source: Clin Exp Med. 2024 Jul 13;24(1):155. doi: 10.1007/s10238-024-01425-w (PMC11246277; doi:10.1007/s10238-024-01425-w)
Supplement: Supplementary file 5 — Supplementary file5 (PDF 550 KB) [file 10238_2024_1425_MOESM5_ESM.pdf]

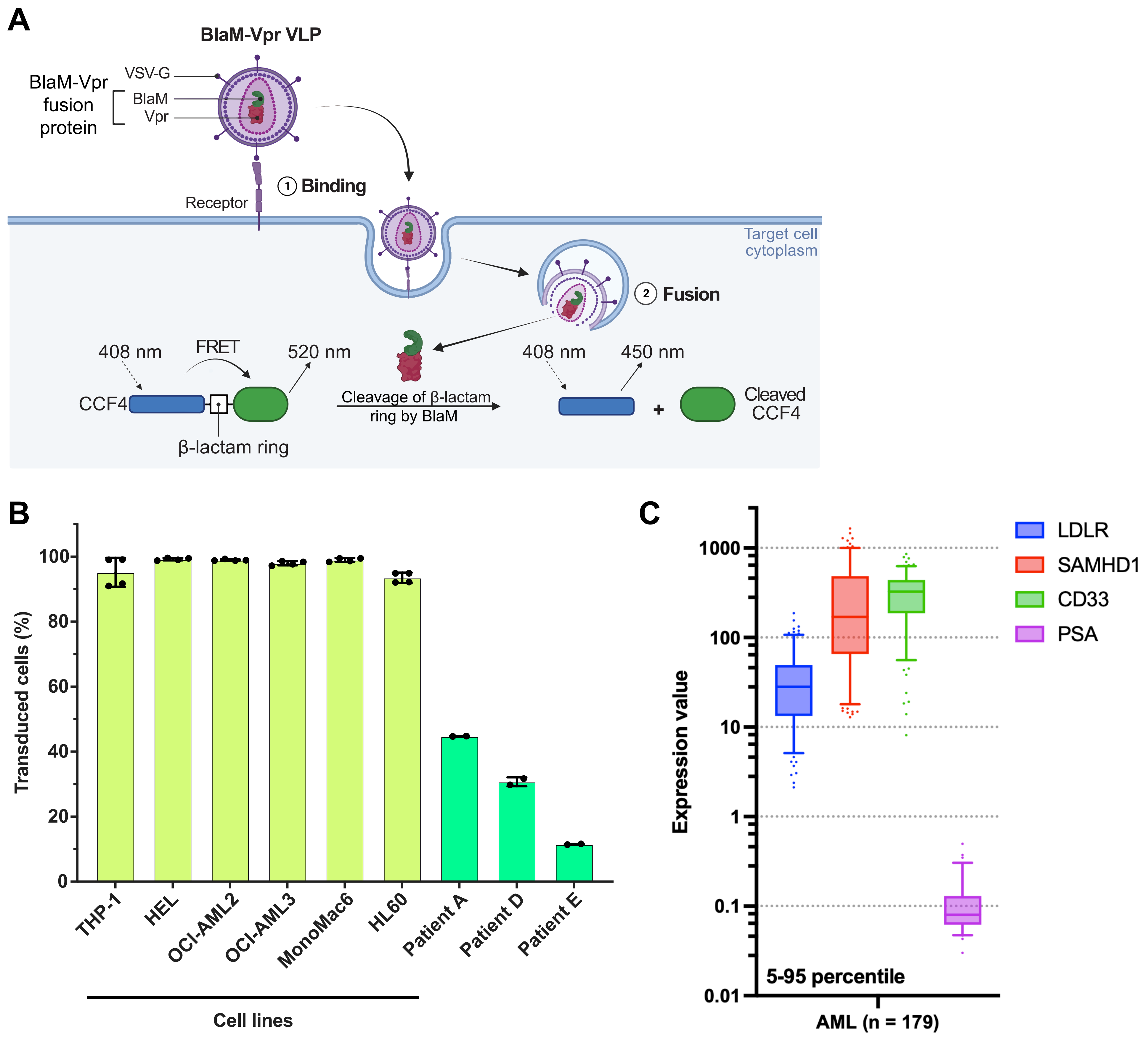

Supplementary Figure 5: Lower fusion efficiency of VSV-G pseudotyped VLPs in AML blasts despite good LDLR mRNA expression levels. A, Schematic of the virion fusion assay based on Cavrois et al. [23]. **B,** AML cell lines and primary AML blasts were transduced with VSV-G pseudotyped Blam-Vpr carrying VLPs. 4h post transduction, cells were stained with CCF4 dye overnight. After fixation with PFA, fusion levels were analyzed by flow cytometry. Shown are arithmetic means ± S.E.M. of 2 independent experiments for cell lines and technical replicates for primary AML blasts. **C,** Shown is the result of TCGA database analysis for LDLR, SAMHD1, CD33, and prostate-specific antigen (PSA) mRNA expression derived from 179 AML patients. PSA served as negative control (PSA expression values equal to 0 were excluded from the logarithmic blot).
