# Supplementary material for: Novel Vpx virus-like particles to improve cytarabine treatment response against acute myeloid leukemia
Source: Clin Exp Med. 2024 Jul 13;24(1):155. doi: 10.1007/s10238-024-01425-w (PMC11246277; doi:10.1007/s10238-024-01425-w)
Supplement: Supplementary file 6 — Supplementary file6 (PDF 119 KB) [file 10238_2024_1425_MOESM6_ESM.pdf]

Supplementary Table 1: Patient characteristics

*ID = initial diagnosis, Fav = favorable, Int = intermediate, Adv = adverse

Patient	Gender	Age	Disease phase	Blasts	Cytogenetics	FAB	ELN (2017)	Mutations
A	Male	77	ID	71%	46, XY	M2	Adv	FLT3-ITD, RUNX1+,
B	Female	33	ID	87%	46, XX	M1	Adv	FLT3-TKD+, NPM1+, IDH1+
C	Female	78	ID	87 %	46, XX	M1	Fav	-
D	Male	82	ID	70%	Complex	-	Fav	FLT3-TKD+
E	Female	56	ID	87 %	Aberrant	M1	Int	KMT2A-PTD
